# Formicarium-Inspired Hierarchical Conductive Architecture for CoSe_2_@MoSe_2_ Catalysts Towards Advanced Anion Exchange Membrane Electrolyzers

**DOI:** 10.3390/molecules30102087

**Published:** 2025-05-08

**Authors:** Zhongmin Wan, Zhongkai Huang, Changjie Ou, Lihua Wang, Xiangzhong Kong, Zizhang Zhan, Tian Tian, Haolin Tang, Shu Xie, Yongguang Luo

**Affiliations:** 1 College of Mechanical Engineering, School of Energy and Electrical Engineering, Hunan Institute of Science and Technology, Yueyang 414006, China; zhongminwan@hnist.edu.cn (Z.W.); 822311110535@vip.hnist.edu.cn (Z.H.); hnlgwlh@163.com (L.W.); zzzhan96@126.com (Z.Z.); 2 State Key Laboratory of Advanced Technology for Materials Synthesis and Processing, Wuhan University of Technology, Wuhan 430070, China; ttcx@whut.edu.cn (T.T.); thln@whut.edu.cn (H.T.); 3 C•HySA Technology (Hunan) Company Limited, Zhuzhou 412007, China; chzhouna@163.com (S.X.); wmluoyg@163.com (Y.L.)

**Keywords:** CoSe_2_@MoSe_2_@NC, formicarium, heteroatom doping, catalytic activity, anion exchange membrane water electrolysis

## Abstract

The exploration of high-performance, low-cost, and dual-function electrodes is crucial for anion exchange membrane water electrolysis (AEMWE) to meet the relentless demand for green H_2_ production. In this study, a heteroatom-doped carbon-cage-supported CoSe_2_@MoSe_2_@NC catalyst with a formicarium structure has been fabricated using a scalable one-step selenization strategy. The component-refined bifunctional catalyst exhibited minimal overpotential values of 116 mV and 283 mV at 10 mA cm^−2^ in 1 M KOH for the hydrogen evolution reaction (HER) and the oxygen evolution reaction (OER), respectively. Specifically, rationally designed heterostructures and flexible carbonaceous sponges facilitate interfacial reaction equalization, modulate local electronic distributions, and establish efficient electron transport pathways, thereby enhancing catalytic activity and durability. Furthermore, the assembled AEMWE based on the CoSe_2_@MoSe_2_@NC bifunctional catalysts can achieve a current density of 106 mA cm^−2^ at 1.9 V and maintain a favorable durability after running for 100 h (a retention of 95%). This work highlights a new insight into the development of advanced bifunctional catalysts with enhanced activity and durability for AEMWE.

## 1. Introduction

With the escalating global demand for clean energy, the development of low-cost, green hydrogen production technologies has emerged as a prominent research frontier. Hydrogen, characterized by its high energy density and zero emissions, is increasingly recognized as a pivotal component in the future energy transition [1]. Presently, the predominant methods for hydrogen production via water electrolysis encompass alkaline water electrolysis (AWE), PEMWE, and AEMWE [2,3]. Among these, AEMWE stands out by integrating the cost-effectiveness of AWE with the high performance and rapid response of PEMWE, positioning it as an ideal candidate for large-scale green hydrogen production in the future [4]. A significant advantage of AEMWE is its compatibility with non-precious metal catalysts in alkaline environments, thereby markedly reducing catalyst costs [5]. Unlike PEMWE, AEMWE obviates the need for precious metals such as platinum (Pt) and iridium (Ir), rendering it more economically viable for extensive applications [6]. The membrane electrode assembly of AEMWE, akin to that of PEMWE, facilitates a swift response and high current density [7]. Typically operating at voltages between 1.8 and 2.5 V, AEMWE effectively segregates hydrogen and oxygen in high-pressure hydrogen production settings, thereby ensuring the safety of the hydrogen generation process [8]. The electrolyte system comprised either deionized water or a 1.0 M aqueous sodium hydroxide solution. AEMWE is environmentally benign, producing no detrimental by-products in the process.

Recent studies on the established porous structures of molybdenum diselenide (MoSe_2_) and cobalt diselenide (CoSe_2_) have led to the synthesis of three-dimensional, highly active catalysts, CoSe_2_/MoSe_2_@CC, grown on a carbon cloth substrate through ion-exchange and gas-phase selenization techniques [9]. This catalyst demonstrated remarkably low overpotentials of 71 mV for the HER and 320 mV for the OER at a current density of 10 mA cm^−2^. Furthermore, CoSe_2_/MoSe_2_@CC exhibited significant catalytic activity for the urea oxidation reaction (UOR) in a 1 M KOH solution containing 0.3 M urea [10]. Building on this, the synthesis of porous CoSe_2_-MoSe_2_ nanosheet arrays via ion exchange and gas-phase selenization has been reported. These catalysts achieved an overpotential of 278 mV and a Tafel slope of 61.2 mV dec^−1^ for OER in a 1 M KOH solution, outperforming commercial RuO_2_ in catalytic activity. Notably, the catalyst’s activity remained virtually unchanged after 24 h of continuous electrolysis. The incorporation of a porous structure has been shown to significantly enhance the electrocatalytic efficiency of MoSe_2_ and CoSe_2_ catalysts, rendering them highly promising for AEMWE applications [11]. Future research endeavors should focus on further optimizing and functionalizing the porous structure to enhance the performance and stability of these catalysts [12]. Such advancements are crucial for promoting the development of low-cost, green hydrogen production technologies, thereby contributing to the global transition towards sustainable energy solutions [13].

In an effort to enhance the electrocatalytic efficiency of CoSe_2_ and MoSe_2_ catalysts, this study synthesized CoSe_2_@MoSe_2_@NC porous structures utilizing the freeze-drying method. Freeze drying serves as a versatile technique for synthesizing porous materials, producing homogeneous pore networks that effectively enhance catalytic activity through increased specific surface area and active site density [14,15]. The synthesis of CoSe_2_ and MoSe_2_ precursors is obtained through chemical precipitation. These precursors were subsequently blended with N-doped carbon (NC) precursors to create a homogeneous slurry. The slurry was then poured into a mold and frozen. The frozen slurry was placed in a freeze dryer and subjected to freeze drying to develop a porous structure. The freeze-dried samples were then subjected to heat treatment under an inert atmosphere to eliminate organic impurities and to finalize the CoSe_2_@MoSe_2_@NC porous structure [16,17]. The porous structure of CoSe_2_@MoSe_2_@NC, prepared by the freeze-drying method, not only markedly increases the specific surface area and the number of active sites of the catalyst but also enhances the efficiencies of charge and mass transport, significantly boosting the electrocatalytic efficiency [18,19]. This porous structure exhibits exceptional performance in AEMWE applications and holds promise for future applications [20]. Future research endeavors should focus on further optimizing the preparation process of the porous structure to enhance the performance and stability of the catalysts, thereby fostering the advancement of low-cost, green hydrogen production technologies [21,22].

## 2. Results and Discussion

**Figure 1:**
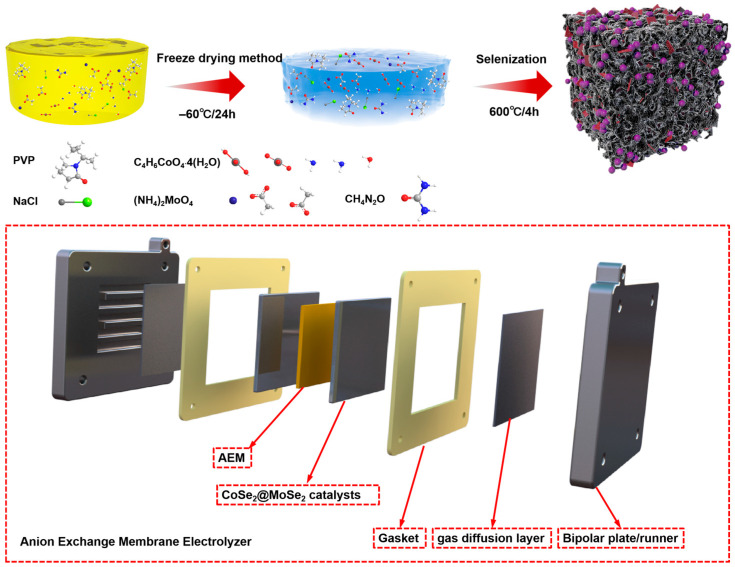
Schematic diagram of CoSe_2_@MoSe_2_@NC synthesis.

Figure 1 is a schematic illustration of the systematic fabrication process for the CoSe_2_@MoSe_2_@NC porous heterostructures. The synthesis commenced with the preparation of composite cobalt-molybdenum metal–organic frameworks (MOFs) through a freeze-drying method, followed by sequential selenization and carbonization processes to construct the hierarchical heterostructure architecture, as detailed in the schematic illustration. Subsequently, the porous CoSe_2_@MoSe_2_@NC composites were fabricated by subjecting the metal–organic frameworks to a high-temperature selenization reaction. This process involves the conversion of the metal–organic precursors into the desired selenide phases, resulting in the formation of the CoSe_2_@MoSe_2_@NC heterostructures with a porous architecture.

**Figure 2:**
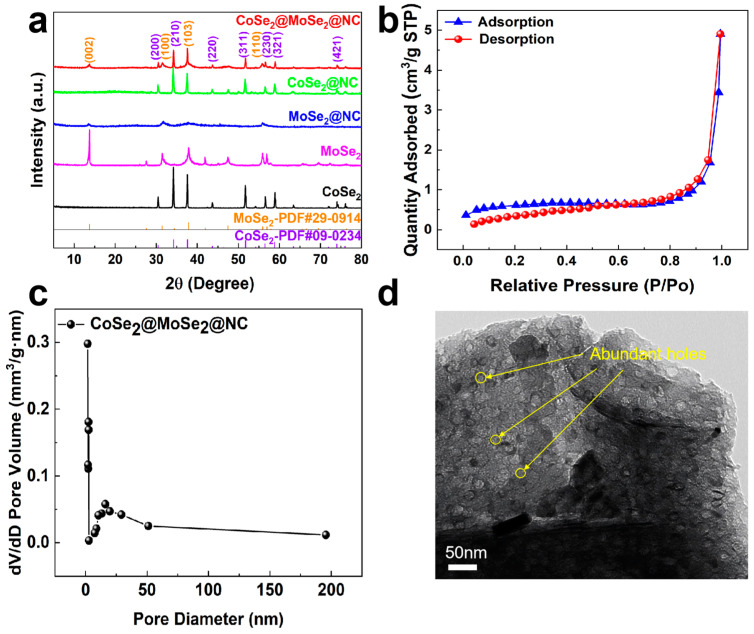
(**a**) Transmission electron microscopy of CoSe_2_@MoSe_2_@NC; (**b**) N_2_ adsorption–desorption diagram of CoSe_2_@MoSe_2_@NC; (**c**) pore size distribution of CoSe_2_@MoSe_2_@NC; (**d**) XRD patterns of CoSe_2_@MoSe_2_@NC and comparison samples.

The phase composition of the samples was meticulously determined using X-ray diffraction spectroscopy (XRD). Figure 2a presents the XRD spectra for CoSe_2_@MoSe_2_@NC, MoSe_2_@NC, MoSe_2_, and CoSe_2_. The diffraction peaks observed for MoSe_2_@NC and CoSe_2_@MoSe_2_@NC at 13.70°, 31.42°, 37.88°, and 55.92° are attributed to the (002), (100), (103), and (110) crystal planes of MoSe_2_, respectively, in accordance with the standard MoSe_2_ (PDF#29-0914). Furthermore, the CoSe_2_@MoSe_2_@NC pattern exhibits additional peaks at 30.48°, 34.20°, 43.69°, 51.75°, 56.48°, 58.85°, and 74.00°, which correspond to the (200), (210), (220), (311), (230), (321), and (421) crystallographic facets of CoSe_2_, respectively, as per JCPDS file no. 09-0234. The XRD spectrum of CoSe_2_@NC, depicted in Figure 2a, reveals a composite profile indicative of both orthorhombic CoSe_2_ (JCPDS 53-0449) and cubic CoSe_2_ (JCPDS 09-0234), suggesting the coexistence of polymorphic phases. The diffraction peaks of the CoSe_2_@MoSe_2_@NC composite align well with the reference patterns for CoSe_2_ (JCPDS 09-0234) and MoSe_2_ (JCPDS 29-0914), confirming the composition of the porous material as a binary mixture of CoSe_2_ and MoSe_2_ without detectable impurities.

To ascertain the pore volumes within these samples, the N_2_ adsorption–desorption technique was utilized. Figure 2b illustrates that the CoSe_2_@MoSe_2_@NC composite manifests type IV isotherms, a hallmark of its mesoporous texture. The specific surface area of the CoSe_2_@MoSe_2_@NC composite was determined to be 2.3 m^2^ g^−1^. A comparison of the specific surface area values with published materials is shown in Table S1. Furthermore, the pore size distribution, as detailed in Figure 2c, is predominantly concentrated within the 2 to 12 nm range, which is indicative of the presence of mesopores [23]. The abundance of mesopores and the elevated specific surface area facilitate comprehensive electrolyte penetration, augment the number of reactive sites, and attenuate the diffusion pathway for hydroxide ions (OH^−^), culminating in superior electrochemical performance.

Investigations into the crystal structure and morphology of the CoSe_2_@MoSe_2_@NC composite were conducted, employing transmission electron microscopy (TEM). The TEM image depicted in Figure 2d reveals an extensive array of uniformly distributed porous structures across the material’s surface. Scanning electron microscopy (SEM) observations have elucidated the intricate morphology of the CoSe_2_@MoSe_2_@NC composites, revealing a hierarchical porous network structure, as depicted in Figure S1. This architectural feature is instrumental in enhancing the electrochemical performance of the material. Figure S1 present SEM images of the CoSe_2_@MoSe_2_@C, CoSe_2_@NC, MoSe_2_@NC, CoSe_2_, and MoSe_2_ individual components, further illustrating the distinct morphological characteristics of each. The hierarchical porous network structure of the carbon skeleton not only provides a multitude of active sites for electrochemical processes but also serves as a robust carrier for two-dimensional MoSe_2_ nanosheets and CoSe_2_ nanocrystals [24]. This synergistic combination of structural elements is anticipated to significantly boost the electrocatalytic activity and stability of the CoSe_2_@MoSe_2_@NC composites in various electrochemical applications.

**Figure 3:**
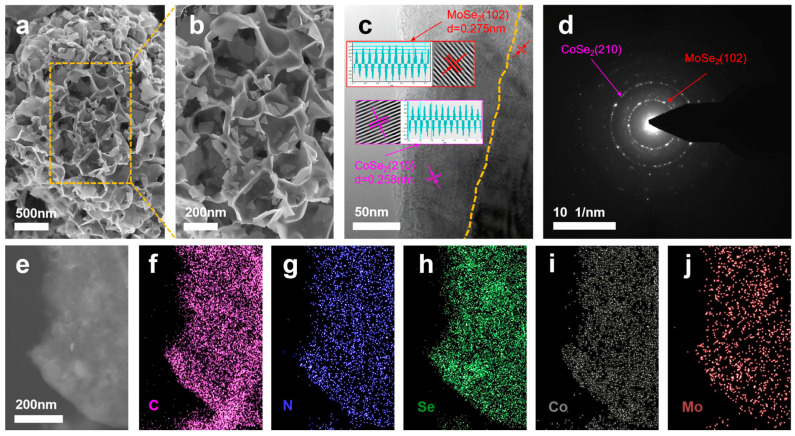
(**a**,**b**) Scanning electron microscopy (SEM) image of the CoSe_2_@MoSe_2_@NC sample. (**c**) High-magnification transmission map of CoSe_2_@MoSe_2_@NC; (**d**) SAED map of CoSe_2_@MoSe_2_@NC; (**e**–**j**) Elemental mapping of CoSe_2_@MoSe_2_@NC.

Figure 3a shows an SEM image of the CoSe_2_@MoSe_2_@NC sample, which had a wave-like nanosheet-like structure. After the simple chemical etching treatment, the morphology of the CoSe_2_@MoSe_2_@NC sample was well maintained (Figure 3b). High-resolution transmission electron microscopy (HRTEM) images, as depicted in Figure 3c, provide compelling evidence that the outer layer of the CoSe_2_@MoSe_2_@NC composite is enveloped by an amorphous carbon matrix. The distinct crystal structures of CoSe_2_ and MoSe_2_ are discernible through their unique stripe spacings of 0.258 and 0.275 nanometers, respectively, which correspond to the (210) and (102) planar distances of the crystal planes. Selected area electron diffraction (SAED) patterns, as shown in Figure 3d, exhibit circular shapes indicative of the CoSe_2_ (321) and (102) planes, underscoring the successful fabrication of heterostructures between CoSe_2_ and MoSe_2_ [25]. The formation of a robust and interface-rich heterostructure between CoSe_2_ and MoSe_2_ is pivotal for accelerating electron transport, thereby enhancing the catalytic activity for water decomposition. Elemental mapping, as illustrated in Figure 3e–j, reveals a relatively uniform spatial distribution of C, N, Se, Co, and Mo, confirming the homogeneous dispersion of CoSe_2_ nanocrystals on the surface of MoSe_2_ nanosheets and their integration with the porous carbon matrix. This uniform distribution is crucial for ensuring efficient electron transfer and catalytic activity across the material [26].

**Figure 4:**
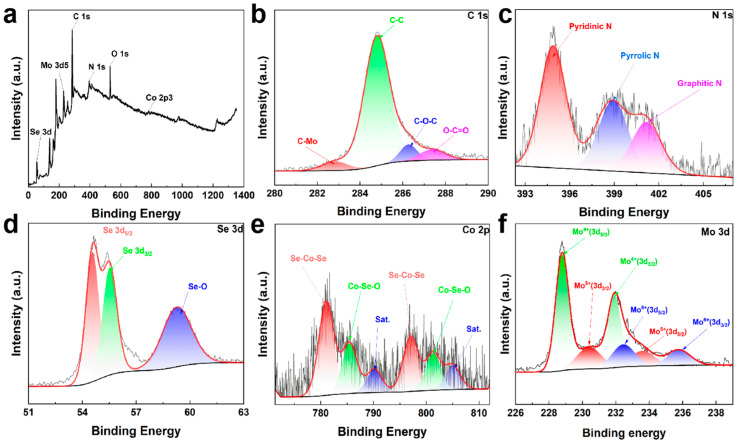
(**a**) XPS spectra of CoSe_2_@MoSe_2_@NC; (**b**–**f**) Fine spectra of C 1s, N 1s, Se 3d, Co 2p, Mo 3d.

X-ray photoelectron spectroscopy (XPS) was employed to elucidate the oxidation states of the constituent elements in the CoSe_2_@MoSe_2_@NC complexes and to investigate the relationship between their conformational structures and properties. The XPS survey scan, as shown in Figure 4a, confirms the presence of the elements C, N, Mo, Co, and Se within the complexes. The high-resolution C 1s spectrum, depicted in Figure 4b, exhibits five distinct peaks at 284.80 eV (C = C), 286.27 eV (C-O-C), 287.38 eV (O = C-O), and 282.83 eV (C-Mo), with the C-Mo bonds suggesting chemical interactions between molybdenum cations and carbon atoms on the surface, potentially resulting from the interaction between ammonium molybdate and PVP during synthesis. The presence of an oxygen phase bond in the C 1s spectrum is attributed to the low selenization temperature used in the process.

Two major peaks within the binding energy range of 392 to 404 eV are identified in Figure 4c, corresponding to Mo 3p_3/2_ and N 1s, respectively. The N 1s spectrum, which is well fitted with three decomposition peaks, is associated with pyridine N (398.38 eV), pyrrole N (398.95 eV), and graphite N (401.17 eV). In the Se spectrum (Figure 4d), a large peak and a smaller peak are visible. The large peak decomposes into two subpeaks, Se 3d_5/2_ (54.56 eV) and Se 3d_3/2_ (55.53 eV). The smaller peak at 59.28 eV is attributed to the Se-O bond, originating from the partial surface oxidation of selenium. The C-Mo bonds are also identified on the Mo 3d spectrum, indicating their presence in the material. The Co 2p spectrum (Figure 4e) displays multiple deconvolution peaks in addition to the satellite spectrum (Sat). These peaks are ascribed to the Co-Se-O and Co-Se bonds of CoSe_2_, with the presence of cobalt selenates likely resulting from the surface oxidation of CoSe_2_. In the Mo 3d spectrum, besides the broad peaks related to C-Mo bonds, typical Mo 3d_3/2_ peaks are observed, as shown in Figure 4f.

**Figure 5:**
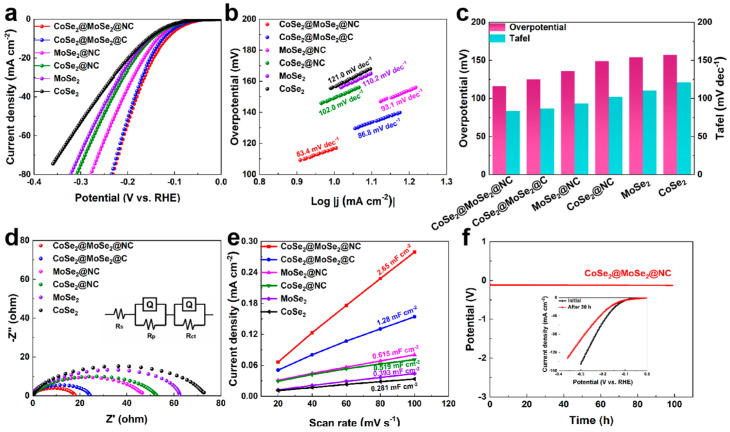
(**a**) LSV polarization curves of HER; (**b**) Tafel plots; (**c**) Overpotential and Tafel comparisons; (**d**) Corresponding Nyquist plots; (**e**) Linear fits to corresponding C_dl_ values; (**f**) Stability plots for CoSe_2_@MoSe_2_@NC (inset: LSV before and after stability test).

A systematic investigation into the electrochemical catalytic activities of CoSe_2_@MoSe_2_@NC, CoSe_2_@MoSe_2_@C, CoSe_2_@NC, MoSe_2_@NC, MoSe_2_, and CoSe_2_ was conducted to assess their potential applications in electrocatalysis, with the results depicted in Figure 5. The electrocatalytic performance of these catalysts was evaluated using linear scanning voltammetry (LSV) in a 1 M KOH electrolyte at a scan rate of 5 mV s^−1^. The LSV curves, as shown in Figure 5a, reveal that the CoSe_2_@MoSe_2_@NC composite, featuring a porous carbon network, exhibits superior HER activity among the tested materials. Specifically, at a current density of 10 mA cm^−2^, the CoSe_2_@MoSe_2_@NC composite demonstrates an overpotential of 116 mV, which is markedly lower than that of CoSe_2_@MoSe_2_@C (125 mV), CoSe_2_@NC (149 mV), MoSe_2_@NC (136 mV), CoSe_2_ (157 mV), and MoSe_2_ (154 mV). This comparative analysis underscores the enhanced electrocatalytic activity of the CoSe_2_@MoSe_2_@NC composite, which can be attributed to its porous carbon network, which facilitates efficient electron transport, optimizes electrical conductivity, and promotes effective interfacial contact with the electrolyte.

The lower overpotential observed for the CoSe_2_@MoSe_2_@NC catalyst indicates that its porous structure significantly enhances electrocatalytic activity by facilitating rapid electron transport, ensuring good electrical conductivity, and promoting effective interfacial contact between the electrode and the electrolyte [27]. Furthermore, the Tafel slope derived from the fitted LSV curves is closely associated with the kinetics of the catalytic reaction. As illustrated in Figure 5b,c, the Tafel slope for the porous carbon network CoSe_2_@MoSe_2_@NC is measured at 83.4 mV dec^−1^. This value is notably lower than those of CoSe_2_@MoSe_2_@C (86.8 mV dec^−1^), CoSe_2_@NC (102.0 mV dec^−1^), MoSe_2_@NC (93.1 mV dec^−1^), CoSe_2_ (121.0 mV dec^−1^), and MoSe_2_ (110.2 mV dec^−1^), suggesting that the intimate contact within the CoSe_2_@MoSe_2_@NC interfacial structure significantly enhances the kinetics of the HER. These findings imply that the synthesized porous material, CoSe_2_@MoSe_2_@NC, offers an increased number of active sites due to the strong interface formed between CoSe_2_ and MoSe_2_, which contributes to the overall enhancement of its electrocatalytic performance. Additionally, XPS results obtained at the electrochemical interface of CoSe_2_@MoSe_2_@NC indicate a favorable electron–donor–acceptor interaction between CoSe_2_ and MoSe_2_, further promoting higher catalytic kinetics.

To verify the charge transfer kinetics at the electrodes, an electrochemical impedance spectroscopy (EIS) analysis was conducted, as illustrated in Figure 5d. The Nyquist plots were fitted to equivalent circuits, with the equivalent circuit model used to represent the impedance data of the catalyst shown in the figure. The EIS fitting parameters are tabulated in Table S2. In this model, Rs and R_ct_ denote the solution resistance and charge transfer resistance, respectively. The varying diameters of the semicircles indicate differences in R_ct_, which can enhance the kinetics of slow reactions. Notably, CoSe_2_@MoSe_2_@NC exhibited the smallest charge transfer resistance (R_ct_) compared to CoSe_2_@MoSe_2_@C, CoSe_2_@NC, MoSe_2_@NC, CoSe_2_, and MoSe_2_. This finding confirms that the favorable interfacial charge transfer characteristics of the selenide heterostructures effectively activate the HER at low overpotentials.

The low R_ct_ values are likely attributed to the overall electronic structure of the catalyst and the open network architecture of CoSe_2_@MoSe_2_@NC, which facilitates mass transport and the release of the evolved H_2_ gas. In addition to the high electrochemically active surface area (ECSA) and the advantageous release of gas due to the structural design, the heterogeneity of the structure endows unique internal electronic attributes that further bolster the catalyst’s HER performance. Generally speaking, the double-layer capacitance (C_dl_) is indicative of the ECSA. The larger the ECSA value, the greater the number of active sites available on the catalyst, which in turn translates to superior electrocatalytic performance.

As depicted in Figure 5e, the calculated C_dl_ value for CoSe_2_@MoSe_2_@NC is 2.65 mF cm^−2^, which is significantly higher than that of CoSe_2_@MoSe_2_@C (1.28 mF cm^−2^), CoSe_2_@NC (0.519 mF cm^−2^), MoSe_2_@NC (0.615 mF cm^−2^), CoSe_2_ (0.281 mF cm^−2^), and MoSe_2_ (0.393 mF cm^−2^). The corresponding ECSA values of the CoSe_2_@MoSe_2_@NC, CoSe_2_@MoSe_2_@C, CoSe_2_@NC, MoSe_2_@NC, CoSe_2_, and MoSe_2_ are calculated to be 132.5 cm^−2^, 64 cm^−2^, 25.95 cm^−2^, 30.75 cm^−2^, 14.05 cm^−2^, and 19.65 cm^−2^, respectively. These increases in C_dl_ value by more than fourfold and sixfold, respectively, suggest that the heterogeneous structure of CoSe_2_@MoSe_2_@NC provides a larger ECSA, thereby conferring a higher level of HER activity compared to that of its bulk counterparts. Furthermore, the durability of the electrodes is a critical consideration for their industrial applicability. A 100 h stability test, as illustrated in Figure 5f, demonstrates that CoSe_2_@MoSe_2_@NC maintains a constant potential with minimal fluctuation at a current density of 10 mA cm^−2^, indicative of its excellent catalytic stability for HER. The LSV curves of the CoSe_2_@MoSe_2_@NC catalysts exhibit an overpotential difference of only 14 mV at 10 mA cm^−2^ before and after the stability tests, further attesting to its robust electrocatalytic activity for HER.

To gain deeper insights into the accelerated reaction kinetics, the ECSA of the catalysts was assessed using cyclic voltammetry (CV) results obtained at varying scanning rates (20–100 mV s^−1^) as shown in Figure S2.

**Figure 6:**
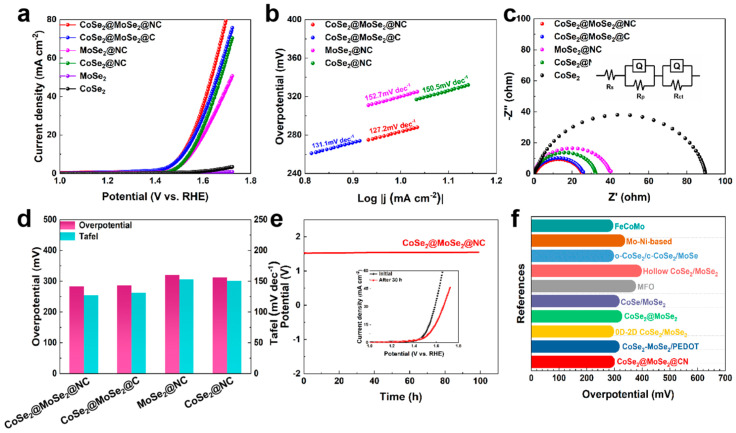
(**a**) LSV curves of OER; (**b**) Tafel curves; (**c**) Nyquist plots; (**d**) Overpotential and Tafel comparison plots; (**e**) Stability plots of CoSe_2_@MoSe_2_@NC (inset: LSVs before and after stabilization); and (**f**) Comparison of OER performances.

We assessed the electrocatalytic activity for the oxygen evolution reaction (OER) of CoSe_2_@MoSe_2_@NC, CoSe_2_@MoSe_2_@C, CoSe_2_@NC, MoSe_2_@NC, CoSe_2_, and MoSe_2_ catalysts in alkaline electrolytes, employing a conventional three-electrode cell setup. As depicted in Figure 6a, the current density escalates sharply with the increment of positive potential, signifying the commendable OER activity of the synthesized electrocatalysts. Specifically, at a current density of 10 mA cm^−2^, the CoSe_2_@MoSe_2_@NC catalyst exhibited an overpotential of 283 mV, outperforming CoSe_2_@MoSe_2_@C (286 mV), CoSe_2_@NC (313 mV), and MoSe_2_@NC (320 mV). Notably, CoSe_2_ and MoSe_2_ failed to achieve a current density of 10 mA cm^−2^.

As presented in Figure 6b,d, the Tafel slope for CoSe_2_@MoSe_2_@NC is a notably lower value of 127.2 mV dec^−1^ compared to CoSe_2_@NC and MoSe_2_@NC, which are 150.5 and 152.7 mV dec^−1^, respectively. This suggests that the OER reaction rate for CoSe_2_@MoSe_2_@NC is more favorable. To elucidate the factors contributing to the enhanced OER performance of CoSe_2_@MoSe_2_@NC, we determined the charge transfer resistance (R_ct_) values of the catalysts from the fitted EIS spectra. CoSe_2_@MoSe_2_@NC exhibited the smallest R_ct_ value, indicative of its favorable OER reaction kinetics, as shown in Figure 6c.

Stability tests on CoSe_2_@MoSe_2_@NC were conducted by applying a constant current of 10 mA, as depicted in Figure 6e. Initially, there is a slight decrease in current density, followed by a gradual stabilization, indicating the good electrocatalytic stability of the CoSe_2_@MoSe_2_@NC structure during the OER process. The inset in Figure 6e displays the LSV curves of CoSe_2_@MoSe_2_@NC before and after the stability test, with an overpotential difference of 50 mV at 10 mA cm^−2^ observed after 30 h of operation. The porous architecture of CoSe_2_@MoSe_2_@NC demonstrated superior electrocatalytic performance compared to previously reported molybdenum-based catalysts, particularly at low overpotentials, as illustrated in Figure 6f. Also, see Table S3 for a comparison of electrochemical properties between our work and other materials.

**Figure 7:**
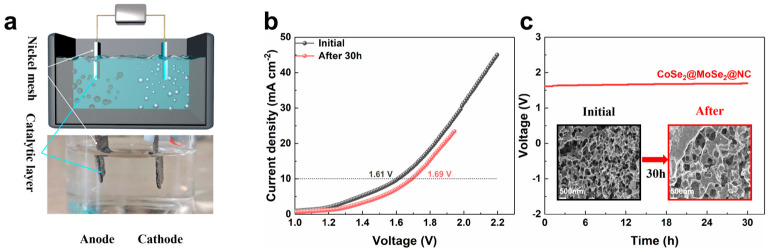
(**a**) Diagram of the fully electrolyzed water reaction setup; (**b**) LSV diagram before and after stability tests; (**c**) Stability diagram of CoSe_2_@MoSe_2_@NC (inset: SEM before and after stability test).

The aforementioned findings reveal that the CoSe_2_@MoSe_2_@NC electrode possesses high electrocatalytic activity for both HER and OER. This prompts a further assessment of its potential as a bifunctional electrocatalyst for overall water splitting in 1.0 M KOH solutions. As observed in Figure 7a, a prolific generation of H_2_ and O_2_ bubbles is evident at the anode and cathode, respectively, during the electrolysis process. Figure 7b compares the LSV values before and after the stability test, indicating that the CoSe_2_@MoSe_2_@NC porous heterostructure requires a cell voltage of 1.61 V to achieve a water decomposition current density of 10 mA cm^−2^. Post-stability tests, the voltage increased by 80 mV at 10 mA cm^−2^, yet remained substantially stable, underscoring the CoSe_2_@MoSe_2_@NC catalyst’s excellent stability in overall water splitting. Furthermore, the bifunctional CoSe_2_@MoSe_2_@NC catalyst electrode sustained a stable water electrolysis performance over a 30 h period, as depicted in Figure 7c. SEM images confirm that the pristine nanostructures of CoSe_2_@MoSe_2_@NC are largely preserved before and after the experiments, as shown in the inset of Figure 7c.

**Figure 8:**
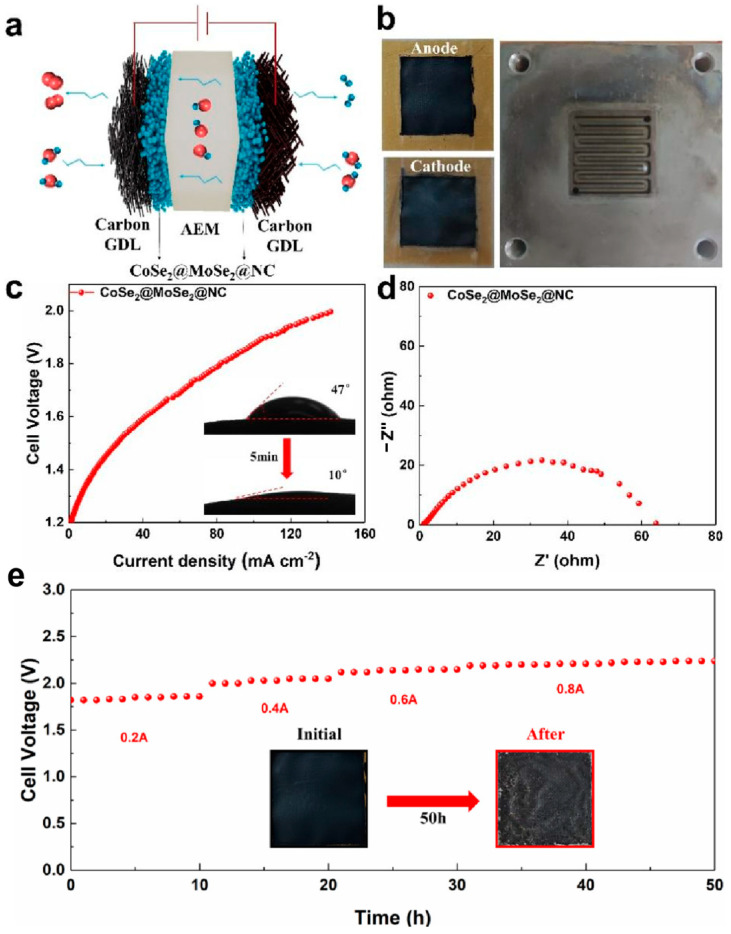
(**a**) Schematic diagram of the AEM reaction; (**b**) Physical diagram of the electrolyzer components; (**c**) Performance diagram of the device (inset: membrane electrode contact angle); (**d**) Impedance diagram of the device; (**e**) Stability diagram of the device (inset: membrane electrode diagram before and after 50 h).

To substantiate its practical implications, membrane electrode assemblies for small-scale hydrogen production via water electrolysis were fabricated by depositing CoSe_2_@MoSe_2_@NC catalysts onto anion exchange membranes. Figure 8a depicts the reaction scheme of an AEM water decomposition device, while Figure 8b presents a physical diagram of the membrane electrodes, encompassing the anode, cathode, and flow field. The device exhibited remarkable performance in alkaline electrolytes, achieving current densities of up to 106 mA cm^−2^ at an applied voltage of 1.9 V, as shown in Figure 8c. The contact angle of the membrane electrode, as shown in the inset of Figure 8c, was measured to be 47° with the electrolyte, diminishing to 10° after a 5 min period. This significant reduction in contact angle underscores the membrane electrode’s exceptional hydrophilicity when coated with the CoSe_2_@MoSe_2_@NC catalyst, ensuring the optimal and thorough absorption of the electrolyte by the membrane. Furthermore, Figure 8d demonstrates that CoSe_2_@MoSe_2_@NC possesses low resistance. The H_2_ and O_2_ gases collected by the device indicated an actual H_2_:O_2_ ratio of approximately 2:1, signifying that the Faradaic efficiency of CoSe_2_@MoSe_2_@NC in facilitating overall water decomposition was nearly 100%, thereby highlighting its high selectivity in electrocatalyzing alkaline water decomposition. Figure 8e illustrates that there was no significant voltage escalation following 50 h of durability testing under varying current conditions. Additionally, the figure shows no signs of cracking on the membrane electrode’s surface after 50 h in the endurance evaluation. These findings underscore that CoSe_2_@MoSe_2_@NC is an exemplary electrocatalyst for alkaline water electrolysis, exhibiting both stability and durability.

## 3. Preparation of Materials

Synthesis of MoSe_2_ and CoSe_2_: Commercially available MoSe_2_ (Aladdin) was used. CoSe_2_ was synthesized via two-step thermal processing: (1) cobalt acetate (2.0 g) and selenium powder (2.0 g) were mixed in a crucible and calcined at 600 °C for 4 h under Ar atmosphere; (2) the product was ground, washed with DI water, and vacuum-dried at 60 °C for 24 h.

CoSe_2_@NC and MoSe_2_@NC composites were synthesized through a three-step process: 10 g polyvinylpyrrolidone (PVP) was dissolved in 100 mL deionized water under stirring, followed by sequential addition of cobalt acetate (2.5 g), sodium chloride (8 g), and urea (5 g) with continuous stirring until dissolution. The solution was freeze-dried (−60 °C, 24 h) and vacuum-dried (48 h), after which the resulting powder was transferred to boats containing 2 g selenium powder, sealed, and selenized at 600 °C (4 h, Ar atmosphere). Post-sintering, the samples were ground, washed with DI H_2_O to remove impurities, and vacuum-dried at 60 °C (24 h). MoSe_2_@NC synthesis followed identical steps, substituting cobalt acetate with ammonium molybdate (2.5 g).

The synthesis of CoSe_2_@MoSe_2_@NC and CoSe_2_@MoSe_2_@C heterostructures: The preparation procedure commenced with the dissolution of 10 g of PVP in 100 mL of deionized water under vigorous stirring to achieve complete dissolution. Subsequently, 2.5 g of cobalt acetate, 2.5 g of ammonium molybdate, 8 g of sodium chloride, and 5 g of urea were introduced into the solution in sequential order, with each component being thoroughly stirred to ensure its dissolution. The resulting solution was then transferred into Petri dishes and subjected to freezing at −60 °C for 24 h, followed by a 48 h drying period in a vacuum oven. The freeze-dried specimens were then each placed in a porcelain boat containing 2 g of selenium powder. These boats were sealed and introduced into a tube furnace, where they were subjected to a selenization process at 600 °C under an argon atmosphere for 4 h. After natural cooling to room temperature, the samples were ground into a fine powder, filtered, and cleaned repeatedly with deionized water to remove any residual impurities. The samples were subsequently dried under vacuum at 60 °C for 24 h to yield the CoSe_2_@MoSe_2_@NC heterostructure [28,29]. It is noteworthy that the synthesis of CoSe_2_@MoSe_2_@NC can be successfully accomplished without the addition of urea, thus providing flexibility in the synthesis protocol [30].

## 4. Conclusions

In summary, we have developed a facile freeze-drying strategy to synthesize porous cobalt-molybdenum bimetallic selenide heterostructures. The optimized CoSe_2_@MoSe_2_@NC catalyst demonstrates exceptional bifunctional electrocatalytic performance, exhibiting low overpotentials of 283 mV (OER) and 116 mV (HER) in alkaline media, coupled with favorable Tafel slopes of 127.2 and 83.4 mV dec^−1^, respectively [31]. Remarkably, an alkaline electrolyzer employing this catalyst as both an anode and cathode achieves a current density of 10 mA cm^−2^ at a reduced cell voltage of 1.61 V, outperforming most reported non-noble metal-based systems [32,33]. When scaled to membrane electrode assemblies, the catalyst enables industrial-level current densities up to 106 mA cm^−2^ at 1.9 V while maintaining structural integrity for over 100 h of operation. This work not only establishes a novel synthetic paradigm for multimetallic chalcogenides but also provides a blueprint for designing high-performance, cost-efficient electrolysis catalysts through hierarchical interface engineering and electronic structure modulation.

## Data Availability

Data are contained within the article or Supplementary Material.

## Supplementary Materials

The following supporting information can be downloaded at: https://www.mdpi.com/article/10.3390/molecules30102087/s1, Figure S1: (a) Scanning electron microscopy of CoSe_2_@MoSe_2_@NC; (b) Scanning electron microscopy of CoSe_2_@MoSe_2_@C; (c) Scanning electron microscopy of CoSe_2_@NC; (d) Scanning electron microscopy of MoSe_2_@NC; (e) Scanning electron microscopy of CoSe_2_; (f) Scanning electron microscopy of MoSe_2_; Figure S2: Corresponding CV plots; Table S1: Comparison of specific surface area values between our work with previous works; Table S2: The EIS fit parameters; Table S3: Comparison of electrochemical properties with other materials.
