# Supplementary material for: Formicarium-Inspired Hierarchical Conductive Architecture for CoSe2@MoSe2 Catalysts Towards Advanced Anion Exchange Membrane Electrolyzers
Source: Molecules. 2025 May 8;30(10):2087. doi: 10.3390/molecules30102087 (PMC12114377; doi:10.3390/molecules30102087)
Supplement: Supplementary file 1 [file molecules-30-02087-s001.zip › molecules-3587640-supplementary.pdf]

Supporting Information

Formicarium-inspired hierarchical conductive architecture for $CoSe_2$@$MoSe_2$ catalysts towards advanced anion exchange membrane electrolyzers

Zhongmin Wan[1], Zhongkai Huang[1], Changjie Ou[1,*], Lihua Wang[1], Xiangzhong Kong[1,*], Zizhang Zhan[1], Tian Tian[2], Haolin Tang[2], Shu Xie[3] and Yongguang Luo[3]

1 College of Mechanical Engineering, School of Energy and Electrical Engineering, Hunan Institute of Science and Technology, Yue yang, 414006, China.

2 State Key Laboratory of Advanced Technology for Materials Synthesis and Processing, Wuhan University of Technology, Wuhan, 430070, China.

3 C•HySA Technology(Hunan) Company Limited, Zhuzhou, 412007, China.

* Corresponding authors: ouchangjie06@163.com, xzhkong@hotmail.com.

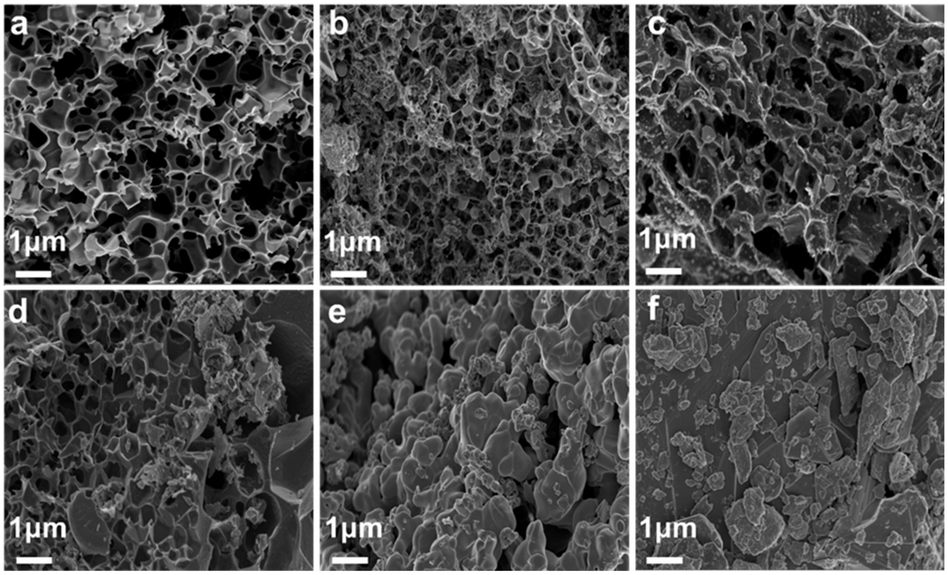

Figure S1. (a) Scanning electron microscopy of $CoSe_2$@$MoSe_2$@NC; (b) Scanning electron microscopy of $CoSe_2$@$MoSe_2$@C; (c) Scanning electron microscopy of $CoSe_2$@NC; (d) Scanning electron microscopy of $MoSe_2$@NC; (e) Scanning electron microscopy of $CoSe_2$; (f) Scanning electron microscopy of $MoSe_2$.

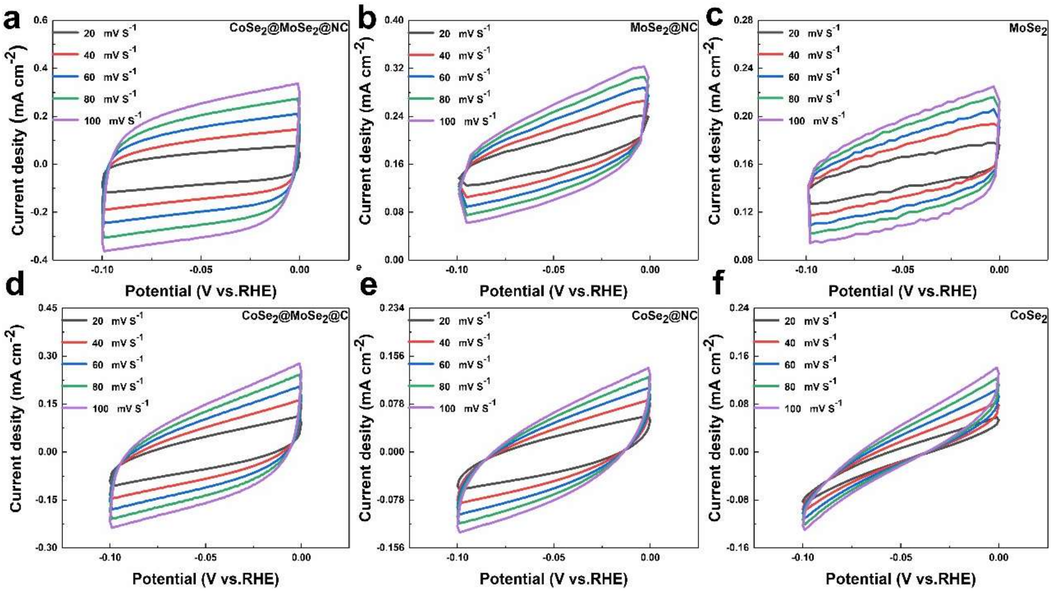

Figure S2. Corresponding CV plots.

Tabol S1. Comparison of specific surface area values between our work with previous works.

Material Description	Specific Surface Area (m²/g)	Reference
$CoSe_2$@$MoSe_2$@NC (this work)	2.3	This work
Hollow $CoSe_2$-$MoSe_2$/Carbon Microspheres	2.5	[19]
3D $CoSe_2$@$MoSe_2$ Core-Shell Heterostructure	3.0	[31]
Se vacancy-engineered $CoSe_2$/$MoSe_2$ Heterostructures	5.8	[25]

Tabol S2. The EIS fit parameters.

Catalyst	Rs (Ω cm^2)	Rct (Ω cm^2)	CPE-T (µF cm^{-2} sP^{-1})	CPE-P	Chi-squared (χ^2)
$CoSe_2$@$MoSe_2$@NC	1.2	3.5	25.6	0.85	1.2×10^{-3}
$CoSe_2$@$MoSe_2$@C	1.5	12.8	18.3	0.78	2.5×10^{-3}
$CoSe_2$@NC	1.8	24.6	12.7	0.72	3.8×10^{-3}
$MoSe_2$@NC	1.6	19.4	15.4	0.75	2.9×10^{-3}
$CoSe_2$	2.1	35.2	8.9	0.68	5.1×10^{-3}
$MoSe_2$	1.9	28.7	10.2	0.70	4.3×10^{-3}

Tabol S3. Comparison of electrochemical properties with other materials.

Catalyst	HER Overpotential (mV@10 mA cm^{-2})	OER Overpotential (mV@10 mA cm^{-2})	Tafel Slope (mV dec^{-1})	Stability (h)	Electrolyte	Ref.
$CoSe_2$@$MoSe_2$@NC	116	283	83.4 (HER), 127.2 (OER)	100 (95% retention)	1 M KOH	This work
$CoSe_2$/$MoSe_2$@CC	71	320	N/A	24	1 M KOH	[9]
$CoSe_2$-$MoSe_2$	N/A	278	61.2 (OER)	24	1 M KOH	[11]
$CoSe_2$-$MoSe_2$/C	98	310	72 (HER), 135 (OER)	50	1 M KOH	[19]
$CoSe_2$/$MoSe_2$@CC (UOR)	N/A	320	N/A	24	1 M KOH + 0.3 M urea	[10]
Pt/C (20 wt%)	~30	N/A	~30	>50	1 M KOH	
IrO_2	N/A	~300	~65	>50	1 M KOH	
RuO_2	N/A	~280	~70			
